# Bromocriptine Does Not Alter Speed–Accuracy Tradeoff

**DOI:** 10.3389/fnins.2012.00126

**Published:** 2012-08-30

**Authors:** Jasper Winkel, Leendert van Maanen, Roger Ratcliff, Marieke E. van der Schaaf, Martine R. van Schouwenburg, Roshan Cools, Birte U. Forstmann

**Affiliations:** ^1^Cognitive Science Center Amsterdam, University of AmsterdamAmsterdam, Netherlands; ^2^Centre for Cognitive Neuroimaging, Donders Institute for Brain, Cognition and Behavior, Radboud University NijmegenNijmegen, Netherlands; ^3^Department of Psychology, Ohio State UniversityColumbus, OH, USA; ^4^Department of Psychiatry, Radboud University Nijmegen Medical CentreNijmegen, Netherlands

**Keywords:** dopamine, speed–accuracy tradeoff, striatum, bromocriptine, functional magnetic resonance imaging, drift diffusion model, linear ballistic accumulator, model-based neuroimaging

## Abstract

Being quick often comes at the expense of being accurate. This speed–accuracy tradeoff is a central feature of many types of decision making. It has been proposed that dopamine plays an important role in adjusting responses between fast and accurate behavior. In the current study we investigated the role of dopamine in perceptual decision making in humans, focusing on speed–accuracy tradeoff. Using a cued version of the random dot motion task, we instructed subjects to either make a fast or an accurate decision. We investigated decision making behavior in subjects who were given bromocriptine (a dopamine receptor agonist) or placebo. We analyzed the behavioral data using two accumulator models, the drift diffusion model, and the linear ballistic accumulator model. On a behavioral level, there were clear differences in decision threshold between speed and accuracy focus, but decision threshold did not differ between the drug and placebo sessions. Bayesian analyses support the null hypothesis that there is no effect of bromocriptine on decision threshold. On the neural level, we replicate previous findings that the striatum and pre-supplementary motor area are active when preparing for speed, compared with accurate decisions. We do not find an effect of bromocriptine on this activation. Therefore, we conclude that bromocriptine does not alter speed–accuracy tradeoff.

## Introduction

Decision making is an essential aspect of everyday life, and the ability to make choices based on available information is a critical function of the brain. One important aspect of making a decision is the speed–accuracy tradeoff (SAT; Wickelgren, 1977): making a decision quickly comes at the expense of being accurate, and vice versa.

**Figure 1:**
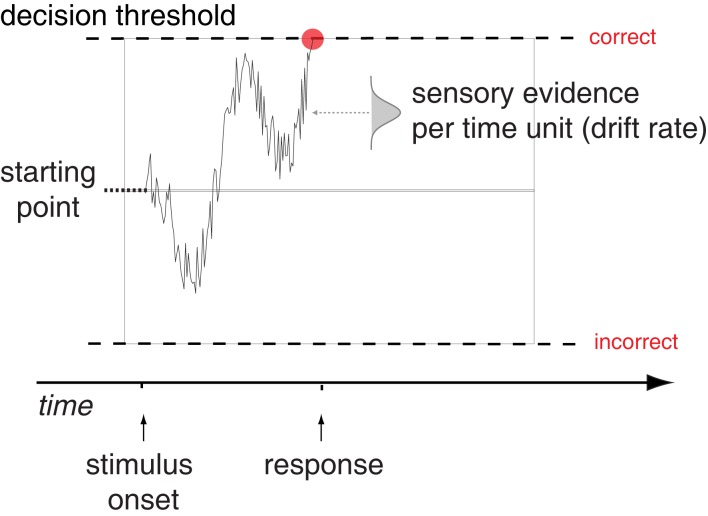
**The Drift Diffusion Model**. Schematic illustration of the main components of the Drift Diffusion Model, showing a sample path for trial where a correct decision is made. Figure adapted from Mulder et al. (2012).

To better understand the underlying processes that generate a decision, several accumulator models of decision making have been formulated. Such models provide a more thorough analysis of decisions than summary statistics, as they explain the entire reaction time distribution of both correct and incorrect responses. These models use several parameters to describe how a decision takes place, and the values for these parameters can be estimated to best explain the behavioral pattern. The prototypical accumulator model is the drift diffusion model (DDM; Ratcliff, 1978), while the Linear Ballistic Accumulator model (LBA; Brown and Heathcote, 2008) is also commonly used. While there are some differences between the two models, the conclusions drawn by these models are largely comparable (Donkin et al., 2011). The DDM explains a decision using information accumulating from a baseline starting point between an upper and a lower threshold (see Figure 1). The distance between the starting point and each threshold represents the amount of information needed to commit to the different response alternatives. As evidence favoring one alternative is collected, the signal accumulates toward the corresponding threshold. When the decision process reaches a threshold, the decision is made. The critical parameter that determines whether the decision emphasizes speed or accuracy is the height of the decision threshold (Ratcliff and Rouder, 1998). A high threshold results in few errors, but slow reactions, whereas a low threshold results in more errors, but faster reactions. The difficulty of the task and the perceptual abilities of the subject are reflected in the drift parameter. An easy task will have a higher drift rate than a hard one, while subjects who are skilled at the task will have higher drift rates than subjects who are not (Ratcliff and McKoon, 2008).

Because of the importance of decision making in cognition, how the brain makes decisions is of great interest to cognitive neuroscience. As such, the neural representation of the parameters that make up a decision has been addressed in many experiments (for reviews, see Gold and Shadlen, 2007; Heekeren et al., 2008; Bogacz et al., 2010). One candidate network to implement the decision threshold is the basal ganglia (BG). The BG are known to play a central role in action selection. According to a common theory, the BG, and more specifically the striatum, select one motor program from a set of competing programs by focally releasing a globally applied inhibition (Mink, 1996). This mechanism makes the BG a likely neural correlate of the SAT element of decision making, by flexibly setting the level of the decision threshold (Lo and Wang, 2006). Recently, research in humans has revealed a frontostriatal network to be associated with speeded responding (Van Veen et al., 2008; Forstmann et al., 2010; Van Maanen et al., 2011; see Bogacz et al., 2010 for a review). Two such studies, using the random dot motion task, showed increased activation in the striatum and pre-supplementary motor area (pre-SMA) when preparing for fast vs. accurate decisions (Forstmann et al., 2008; Van Maanen et al., 2011). Another study manipulated SAT while subjects performed the Simon task (Van Veen et al., 2008). In preparation for fast vs. accurate decisions, the authors find increased sustained baseline activity in a number of regions including the pre-SMA, the striatum, and the intraparietal sulcus (IPS). This sustained baseline activity is associated with reduced transient, event-related activity in the same areas, consistent with the notion that an increased baseline activity requires less additional activation to reach a decision threshold.

One way that the lowering of the threshold might take place is through modulation of striatal neurons by dopamine. Although dopamine is most commonly associated with reward processing, there is a growing body of evidence indicating that dopamine is involved in other aspects of cognition. A neural network model of the cortex and the BG looks into the mechanism for adapting response thresholds during SAT (Lo and Wang, 2006). This model proposes that the strength of corticostriatal synapses determines the height of the decision threshold, setting the level of cortical activation that is required to achieve a response. As the level of corticostriatal connectivity is affected by dopaminergic innervation, this model would predict an important role for dopamine in SAT. Aside from influencing synaptic plasticity in the longer term, dopamine also acts as an immediate neuromodulator, changing the responsivity of striatal neurons on a shorter timeframe. This would suggest that fluctuations in dopamine should result in changes in decision threshold. Other research points toward a similar role for dopamine. Some authors have proposed that dopamine affects behavioral activation (Robbins and Everitt, 2007), while a model of behavioral choice suggests that the tonic level of dopamine acts as a general indicator of response vigor (Niv et al., 2006). Also consistent with a dopaminergic hypothesis of SAT is the finding that ADHD patients have a deficit in setting their decision threshold (Mulder et al., 2010), while the dopamine system is known to be involved in the neuropathology of ADHD (cf. Genro et al., 2008). In a similar vein, patients with Parkinson’s disease have more difficulty making fast responses than healthy controls in both interference (Wylie et al., 2009) and limb movement (Mazzoni and Hristova, 2007) tasks. Based on these findings, we hypothesize that dopamine regulates SAT by increasing striatal excitability, thus lowering the decision threshold.

In the present study, we examine the frontostriatal speed related network using fMRI. We first confirm our previous hypothesis that the striatum and the pre-SMA are involved in setting a threshold for perceptual decision making. Thus, we replicate the finding that the striatum and pre-SMA show increased activation while preparing for fast vs. accurate decisions. Second, we test our main hypothesis that dopamine regulates the decision threshold. We manipulate the dopaminergic system through the partially selective D2/D1 dopamine receptor agonist bromocriptine. This results in deactivation of neurons expressing D2 receptors, such as in the indirect pathway, and to a lesser extent in activation of neurons expressing D1 receptors, such as in the direct pathway. We expect to see changed decision thresholds under bromocriptine, possibly as a function of individual differences in working memory capacity (Kimberg et al., 1997; Cools et al., 2008) or impulsiveness (Cools et al., 2007). We expect to find that the differences in threshold will be accompanied by altered activation in the previously described frontostriatal speed related network in the brain (Forstmann et al., 2008).

## Materials and Methods

### Subjects

Twenty subjects (10 female, age μ = 23.6, σ = 4.3) were recruited from the Nijmegen student population. All subjects gave written informed consent and were compensated for participation. The study was approved by the local ethics committee (committee for the protection of human subjects of the Arnhem/Nijmegen region; CMO protocol number 2008/078).

### Procedure

The experiment took place over the course of three sessions. During the intake session, participants were screened by a medical doctor. This screening included a Mini-International Neuropsychiatric Interview to exclude (a history of) psychiatric diseases (Sheehan et al., 1998). Additionally, an anamnesis and physical examination (weight, heart rate, blood pressure, and electrocardiogram) were completed to exclude relevant medical history, substance abuse, or a family history of psychiatric diseases. Self-report questionnaires and neuropsychological tests were administered to assess personality traits, IQ, and baseline working memory capacity. All scores were within normal range. Finally, the SAT task was practiced in the MRI scanner during acquisition of the structural scans. Subjects were instructed not to use any drugs in the week prior to the experimental sessions, and not to consume any alcohol 24 h prior to either session.

The second and third sessions were performed identically to each other, except that the subject received a placebo in one session and bromocriptine (Parlodel^®^, Novartis, 1.25 mg) in the other. This dose was selected based on previous and similar studies, revealing good tolerance (Gibbs and D’Esposito, 2005; Cools et al., 2009; Van der Schaaf et al., in preparation, Van Schouwenburg et al., in submission). The order of drug application was determined in a double-blind counterbalanced manner. This counterbalancing was performed separately for men and women.

During the second and third sessions, subjects ingested their capsule (bromocriptine or placebo) with a glass of milk at 1.50 p.m. They were asked to wait in an emotionally neutral environment until being escorted to the scanner. The SAT experiment was performed during fMRI acquisition from 3.30 p.m. onward. After this, the subject filled out several state questionnaires and performed another behavioral experiment, which will be reported in a separate paper. These questionnaires were the State Anxiety Inventory (Spielberger et al., 1970; Van der Ploeg et al., 1980), the Barratt Impulsiveness Scale (Patton et al., 1995), the Behavioral Inhibition/Behavioral Activation Scale (BIS/BAS; Carver and White, 1994), and the Positive and Negative Affect Scale (Watson et al., 1988). Background neuropsychological tests assessed at the end of each session day included the digit span test (Groth-Marnat, 1997), a paper and pencil block completion and number cancelation test, and a letter fluency test.

### Experimental paradigm

Subjects performed a random dot motion task in which a cue indicated a trial’s speed or accuracy focus (Forstmann et al., 2008). Subjects responded to a random dot motion stimulus with a left or right hand button press, and were instructed to perform each trial either quickly or accurately depending on the cue. During both sessions, the task comprised two blocks of 105 trials, each consisting of 50 speed, 50 accuracy, and five dummy trials. Every trial onset was locked to each fifth scanner pulse, resulting in a 10 s trial length, regardless of the trial’s RT. All trials started with a fixation cross, presented for 500 ms, followed by a jittered interval (with a duration of 0, 500, 1000, or 1500 ms). After that interval, a cue was presented for 4800 ms. The cue could be either SN for speed focus, or AC for accuracy focus. The cue was followed by a second jittered interval, the length of which compensated for the first jitter so that the sum of the two intervals was always 1500 ms. Next was the random dot motion stimulus with a coherence of 50%, which was presented for 1500 ms, or until a response was made. After a 300 ms delay, the subjects were presented with feedback for 350 ms. For the accuracy trials this feedback could be either “correct” or “incorrect.” For the speed trials this feedback could be either “in time” (when subjects responded before 400 ms) or “too late” (when subjects responded between 400 and 1000 ms). If the subject did not respond within the first 1000 ms of the stimulus, the subject received a feedback stating “no response.” Following feedback presentation, no more stimuli were presented until the start of the next trial. During the dummy trials, a fixation cross was presented for 10 s.

### Bayesian *t*-tests

In several statistical analyses, we report Bayesian posterior probabilities in addition to conventional *p*-values to support the null hypothesis that the behavior during drug and placebo was the same. When we assume, for fairness, that the null hypothesis and the alternative hypothesis are equally plausible *a priori*, a default Bayesian *t*-test (Wetzels et al., 2009) allows one to determine the *posterior* plausibility of the null hypothesis and the alternative hypothesis. We denote the posterior probability for the null hypothesis as *p*^Bayes^(*H*_0_). When, for example, *p*^Bayes^(*H*_0_) = 0.9, this means that the plausibility for the null hypothesis has increased from 0.5 to 0.9, and the plausibility of the alternative hypothesis has correspondingly decreased from 0.5 to 0.1. We report these posterior probabilities because they address several problems both with conventional *p*-values and with *p*_rep_ (Wagenmakers, 2007; Iverson et al., 2008a,b). Most importantly, posterior probabilities allow one to directly quantify evidence in favor of the null hypothesis, instead of only “failing to reject” it. In the case of our analyses, we perform a one-sample Bayesian *t*-test on the difference scores of two measures (during drug and during placebo), because we want to show the posterior probability that they are the same.

### Behavioral analyses

Data from three subjects were excluded due to poor behavioral performance (accuracy <60% in the accuracy condition). Accordingly, we report behavioral data from 17 subjects (8 female; age μ = 23.0, σ = 3.2). We analyzed subjects’ mean RT and accuracy using SPSS (PASW Statistics 18.0 for MacOS). Trials in which there was no response were excluded from the RT calculations. RT and accuracy rates were entered into separate repeated measures ANOVAs with within-subjects factors drug (bromocriptine vs. placebo) and cue (speed vs. accuracy).

Additionally, we analyzed the full correct and incorrect reaction time distributions per subject using two separate accumulation models, the DDM and the LBA model. To model the data, trials with left and right moving dots were collapsed. For each subject, 5 RT quantiles (0.1, 0.3, 0.5, 0.7, 0.9) were computed separately for correct and error responses. A SIMPLEX minimization routine (Nelder and Mead, 1965) was used to optimize the fit of the model predictions’ RT quantiles to those of the behavioral data (Ratcliff and Tuerlinckx, 2002; Brown and Heathcote, 2008).

To model the data with the DDM, we allowed the threshold and non-decision time parameters to vary between the speed and the accuracy condition (e.g., Rinkenauer et al., 2004). The drug and placebo sessions were fit independently, minimizing the chi-square statistic.

To model the data with the LBA model, we defined a single model which allowed the threshold parameter to vary across both condition and session, and fit this to the data using maximum likelihood estimation.

We examined drug effects on threshold using a repeated measures ANOVA with threshold as the dependent measure, and session and condition as within subject measures. Additionally, we performed paired sample *t*-tests and Bayesian *t*-tests, comparing speed and accuracy thresholds between drug and placebo.

In addition to testing for an effect of session on behavioral measures across the group, we also tested whether these effects varied as a function of subjects’ BIS, BAS, or Barratt scores. We computed correlations between these three measures and the difference value of RT and accuracy between the drug and placebo sessions, as computed separately for both the S and the A trials. This resulted in 12 comparisons (2 behavioral measures × 2 conditions × 3 personality measures), giving a Bonferroni corrected alpha of 0.05/12 = 0.004).

### MRI data acquisition

Whole-brain imaging was performed on a 3 T MR scanner (Magnetom Trio Tim, Siemens Medical Systems, Erlangen, Germany). Functional data were obtained using a gradient-echo echo-planar scanning sequence with blood oxygenation level-dependent (BOLD) contrast (30 axial-oblique slices acquired in interleaved order, repetition time = 2000 ms, echo time = 30 ms, voxel size = 3.5 mm × 3.5 mm × 3.0 mm, inter slice gap = 0.5 mm, field of view = 224 mm, flip angle = 80°). Visual stimuli were projected on a screen and were viewed through a mirror attached to the head coil. In addition, a high-resolution T1-weighted MP-RAGE anatomical scan was obtained from each subject (192 sagittal slices, repetition time = 2300 ms, echo time = 3.03 ms, voxel size = 1.0 mm × 1.0 mm × 1.0 mm, field of view = 256 mm).

### MRI preprocessing

The imaging data from three subjects were excluded due to abrupt motion artifacts consisting of translations >6 mm or data acquisition problems. Accordingly, we report imaging data from 14 subjects (6 female, age μ = 22.7, σ = 3.3).

Preprocessing was performed using FSL (FMRIB’s Software Library, version 4.8, www.fmrib.ox.ac.uk/fsl, Smith et al., 2004; Woolrich et al., 2009). The first four volumes of functional data were removed to allow T1 equilibrium to set in. Functional images were corrected for slice time acquisition, pre-whitened, and realigned to compensate for small head movements (Jenkinson et al., 2002). Data were spatially smoothed using a 5 mm full-width-half-maximum Gaussian kernel, and temporally filtered using a high-pass filter with a cutoff time of 80 s, to correct for baseline drifts. Functional images were coregistered to the subject’s T1 structural image, and then normalized to Montreal Neurological Institute (MNI) space using parameters estimated using affine transformation based on the structural image (Jenkinson and Smith, 2001).

### MRI analysis

Functional analysis was performed using FEAT (FMRI Expert Analysis Tool, Version 5.98, part of FSL). Our first level GLM analysis included regressors for the speed and accuracy cues, which were convolved with a double-gamma hemodynamic response function (HRF) and its first-order derivative. The resulting statistical maps were averaged over the two blocks of the same session.

We performed several higher level analyses using FLAME (FMRIB’s Local Analysis of Mixed Effects, Beckmann et al., 2003; Woolrich et al., 2004). These analyses were thresholded at *Z* = 2.3 on the voxel level, and then thresholded at a cluster level (multiple comparisons corrected) *p* value of 0.05. To verify that bromocriptine affected the neural data, we first examined the general effect of drug on the brain, by computing the difference between the effect of any cue vs. implicit baseline during the drug and placebo sessions, regardless of trial type. For the drug and placebo sessions separately, we computed the difference between activation during the speed and accuracy cues (S-A contrast). We also computed covariance analyses for drug and for placebo, using the individual threshold difference between cues (as estimated by the DDM) as a covariate. Second, we examined whether the S-A contrast was modulated by drug, by computing the difference between speed (drug) and speed (placebo), and the difference between accuracy (drug) and accuracy (placebo). To allow us to make statistical inferences regarding activation during drug vs. placebo (Nieuwenhuis et al., 2011), we directly compared effects of drug on the S-A contrast in six regions of interest (ROIs) using a paired samples *t*-test and Bayesian *t*-test (Wetzels et al., 2009). We selected these ROIs based on *a priori* expectations generated by previous research (Forstmann et al., 2008), and defined them anatomically. These six ROIs correspond to the (bilateral) caudate nucleus, putamen, and pre-SMA. We used the Harvard-Oxford Subcortical Structural Atlas within FSL to define the caudate nucleus and putamen ROIs, and anatomical masks ranging from *Y* = 0 to *Y* = 30 to define the pre-SMA ROIs (Johansen-Berg et al., 2004).

Based on the results of these analyses, we needed to get an estimate of the drug effect on the speed network. Directly computing the difference between drug and placebo based on functional ROIs from either of these conditions would produce biased statistics, since it would include those voxels that were significantly active in one condition, even if this were due to noise (Vul et al., 2009). In order to gain a qualitative comparison of subthreshold activations during drug and placebo, we also computed statistical maps without a cluster threshold. In this analysis we used a *Z*-threshold of 2.3, and no cluster-based threshold.

## Results

### Behavioral results

**Figure 2:**
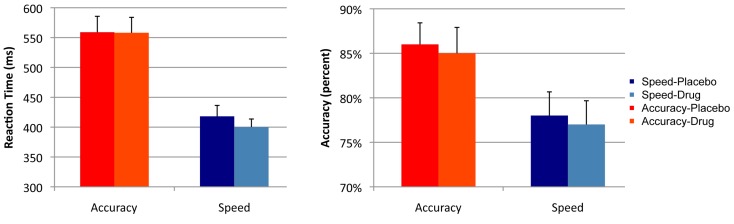
**Summary statistics**. Mean reaction times and accuracy rates across the two sessions (drug and placebo) and the two cues (speed and accuracy). Error bars indicate the standard error of the mean.

Analysis of RT showed a main effect of cue [*F*(1, 16) = 45.2, *p* < 0.001]. There was no effect of drug [*F*(1, 16) = 0.53, *p* = 0.475], and no interaction effect of cue × session [*F*(1, 16) = 1.284, *p* = 0.247]. Analysis of accuracy showed a main effect of cue [*F*(1, 16) = 20.5, *p* < 0.001]. There was no effect of session [*F*(1, 16) = 1.5, *p* = 0.231] and no interaction effect of cue × session [*F*(1, 16) = 0.120, *p* = 0.734; see Figure 2].

Direct comparison of session effects on RT and accuracy did not show significant differences, and the result of the Bayesian *t*-tests showed evidence in favor of the null hypothesis that drug did not affect RT and accuracy. There was no effect of session on RT in the speed [*t*(16) = 1.6, *p* = 0.129; *p*^Bayes^(*H*_0_) = 0.613], or in the accuracy condition [*t*(16) = 0.07, *p* = 0.945; *p*^Bayes^(*H*_0_) = 0.845]. Similarly, there was no effect of session on accuracy in the speed [*t*(16) = 1.07, *p* = 0.296, *p*^Bayes^(*H*_0_) = 0.793] or in the accuracy condition [*t*(16) = 1.182, *p* = 0.254, *p*^Bayes^(*H*_0_) = 0.795].

Analyzing the threshold parameters from the DDM showed a significant effect of condition [*F*(1, 16) = 27.5, *p* < 0.001], but no significant effect of session [*F*(1, 16) = 0.0955, *p* = 0.76], and no interaction effect [*F*(1, 16) = 0.82, *p* = 0.38]. There was a significant difference between the speed and accuracy condition, both during placebo [*t*(16) = 4.31, *p* < 0.001], and during drug [*t*(16) = 3.84, *p* = 0.0014].

Direct comparison of session effects on threshold did not show significant differences, and the result of the Bayesian *t*-tests showed evidence in favor of the null hypothesis that drug did not affect thresholds. There was no effect of session on threshold in the accuracy [*t*(16) = −0.71, *p* = 0.49; *p*^Bayes^(*H*_0_) = 0.81] or in the speed condition [*t*(16) = 0.52, *p* = 0.61; *p*^Bayes^(*H*_0_) = 0.83].

Analyzing the threshold parameters from the LBA model showed a significant effect of condition [*F*(1, 16) = 51.5, *p* < 0.001], but no significant effect of session [*F*(1, 16) = 0.120, *p* = 0.734], and no interaction effect [*F*(1, 16) = 0.025, *p* = 0.877]. There was a significant difference between the speed and accuracy condition, both during placebo [*t*(16) = 6.398, *p* < 0.001], and during drug [*t*(16) = 6.945, *p* < 0.001].

Direct comparison of session effects on threshold did not show significant differences, and the result of the Bayesian *t*-tests showed evidence in favor of the null hypothesis that drug did not affect thresholds. There was no effect of session on threshold in the accuracy[(*t*(16) = −0.12, *p* = 0.91; *p*^Bayes^(*H*_0_) = 0.84] or in the speed condition [*t*(16) = −1.0, *p* = 0.34; *p*^Bayes^(*H*_0_) = 0.77].

**Figure 3:**
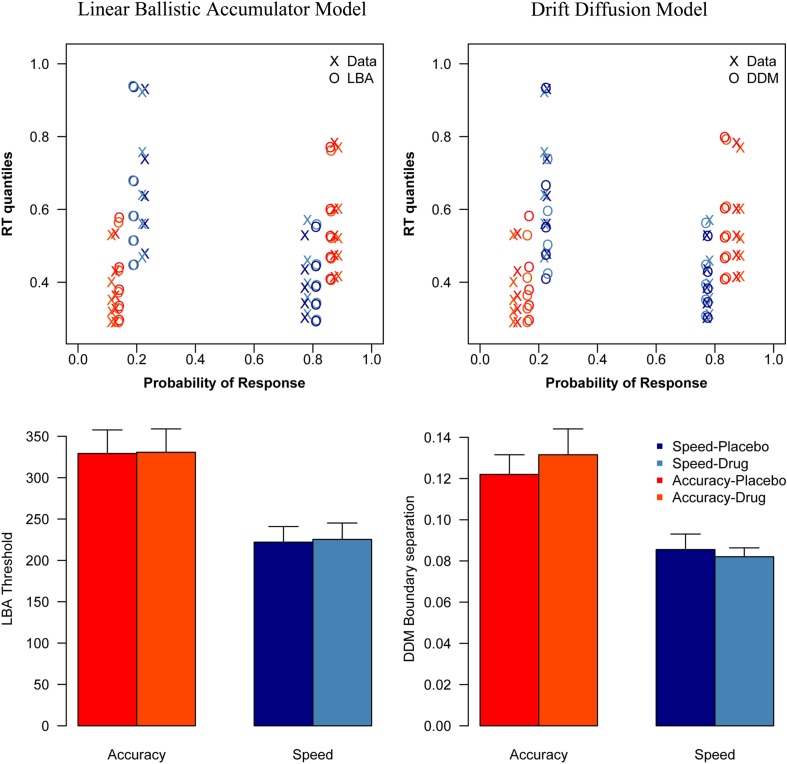
**Model fits and parameter estimates**. This figure shows the vincentized behavioral data and model fits (top) and the threshold estimates per experimental condition (bottom) of the LBA model (left) and the DDM (right). As the reaction time distributions in the top figure are plotted by their probabilities, the error distributions are shown on the left side of each plot, while the correct distributions are shown on the right side.

The DDM and LBA model fits, as well as the corresponding parameter estimates are shown in Figure 3.

The correlations between drug effect on behavior and personality questionnaires did not yield significant activations even before correcting for multiple comparisons. The highest correlation found in these 12 analyses was *R*(17) = 0.482, *p* = 0.051 between subjects’ BIS score and the drug effect on their accuracy in the speed condition. Note that the Bonferroni correction prescribes an alpha value of 0.05/12 = 0.004.

### fMRI results

Our general analysis of the effect of drug on cue (regardless of cue type) vs. implicit baseline revealed significantly activated clusters in the bilateral midbrain (including regions of the brainstem, pallidum, thalamus, and subthalamic nucleus), in the left lateral occipital cortex (including area MT) and in the right inferior frontal gyrus.

**Figure 4:**
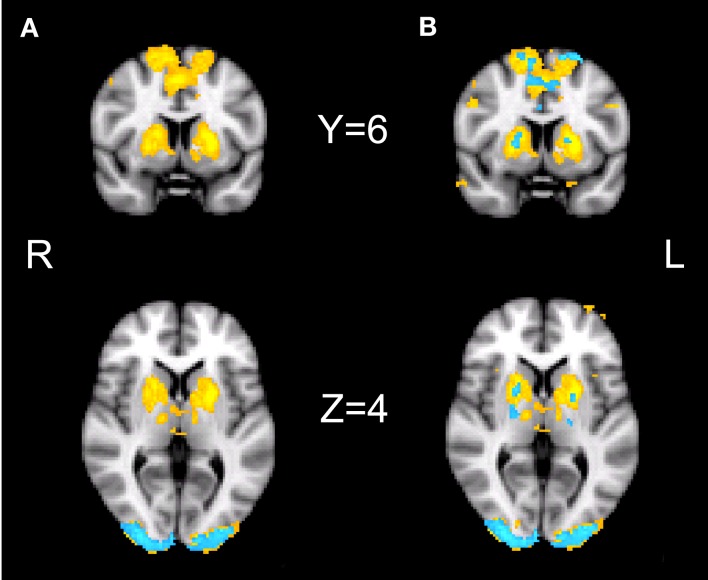
**fMRI results**. Results of the fMRI analyses showing the speed–accuracy contrast during placebo (yellow) and during drug (blue). **(A)** Cluster thresholded with a voxel thresholded of *Z* = 2.3, and a cluster threshold of *p* = 0.05. **(B)** Voxel thresholded results, with a voxel threshold of *Z* = 2.3 and no cluster threshold. The statistical maps are overlaid on Montreal Neurological Institute (MNI) T1 anatomical scans with a 2 mm resolution.

Our initial contrast comparing S-A trials during placebo revealed increased activation in the bilateral striatum, the bilateral pre-SMA, bilateral occipital poles, the right IPS, and the right posterior cingulate cortex. During drug, significant activation was found only in the occipital pole (see Figure 4A; Table 1). However, the analysis of the interaction between session and condition did not yield significant activations at the whole-brain level.

**Table 1:** **fMRI results**.

Region	Cluster size	Cluster *p*	*x*	*y*	*z*
**MAIN EFFECT OF DRUG ON CUE**					
Bilateral brainstem	533	0.00389	0	−15	−8
Right inferior frontal gyrus	459	0.0102	49	24	12
Left lateral occipital cortex	391	0.0257	−44	−70	13
**SPEED–ACCURACY (PLACEBO)**					
Bilateral pre-SMA	3412	1.15e–11	8	7	51
Bilateral striatum	3055	9.87e–11	−2	−3	6
Right occipital pole	1388	7.33e–6	−29	−93	3
Left occipital pole	913	0.000381	29	−97	2
Posterior cingulate	538	0.0145	1	−24	29
Right intraparietal sulcus	448	0.0384	60	−45	39
**SPEED–ACCURACY (DRUG)**					
Left occipital pole	983	0.00115	26	−98	1
Right occipital pole	836	0.00351	−25	−99	3

*Significantly activated clusters (cluster-corrected p < 0.05) in the speed–accuracy contrast during placebo and during drug, based on voxels that exceed a *Z*-threshold of 2.3. The cluster size is in voxels. Coordinates correspond to the spatial center of gravity of the *Z*-values in the cluster, represented in MNI space. pre-SMA, pre-supplementary motor area*.

Direct comparisons of extracted activation levels from caudate nucleus, putamen, and pre-SMA ROIs revealed no significant differences between the drug and the placebo session [left caudate nucleus(*t*(13) = 1.096, *p* = 0.293; *p*^Bayes^(*H*_0_) = 0.74], right caudate nucleus[*t*(13) = 1.293, *p* = 0.219; *p*^Bayes^(*H*_0_) = 0.70], left putamen [*t*(13) = 1.358, *p* = 0.198; *p*^Bayes^(*H*_0_) = 0.68], right putamen [*t*(13) = 1.211, *p* = 0.247; *p*^Bayes^(*H*_0_) = 0.72], left pre-SMA [*t*(13) = 0.842, *p* = 0.415; *p*^Bayes^(*H*_0_) = 0.78], right pre-SMA [*t*(13) = 1.216, *p* = 0.246; *p*^Bayes^(*H*_0_) = 0.72]. Our voxel thresholded qualitative comparison (see Figure 4B) illustrates the presence of subthreshold activation during drug, in the regions that were significantly activated during placebo. The covariate analyses did not yield significant activations in either the drug or placebo conditions.

## Discussion

In this experiment, we have investigated the effect of bromocriptine on speed–accuracy tradeoff. Contrary to our hypothesis, we have found that bromocriptine does not alter decision thresholds in perceptual decisions. There are several possible explanations why we did not find such an effect. We can rule out the explanation that our pharmacological manipulation was unsuccessful, because we do find effects of bromocriptine on neural activations in our main contrast of task. Also, the same dosage and timing schema have been successfully employed to show significant effects in numerous other studies (Cools et al., 2007, 2009; Van Holstein et al., 2011; Van der Schaaf et al., in preparation; Van Schouwenburg et al., in submission). However, we cannot rule out that a higher dose of bromocriptine or a different drug might still affect SAT, for instance because SAT could be mainly driven by D1 receptors, or be sensitive to dopamine precursors or reuptake inhibitors, but not to direct agonists.

The most obvious explanation for our findings is simply that, contrary to our hypothesis, dopamine does not regulate SAT. This interpretation is supported by our Bayesian analyses, which support the null hypothesis that bromocriptine does not affect mean RT and accuracy. Bayesian analyses on the threshold parameters as determined by the DDM and LBA model also support the null hypothesis. However, we should be cautious to draw general conclusions about the role of dopamine based on just these findings using bromocriptine. It is still possible that dopamine does play a role in SAT, but that the relevant aspect of the dopaminergic system is not affected by bromocriptine. This could be due to the receptor specificity of bromocriptine, or due to selective effects of tonic vs. phasic dopaminergic signaling. It could also be the case that decision threshold is less sensitive to this manipulation, requiring a higher dose to be effective. Another possibility is that the postsynaptic effect of bromocriptine is counteracted by its effect on presynaptic autoreceptors, which might decrease the amount of dopamine released into the synapse (Laakso et al., 2005; Stelzel et al., 2010). Further experiments using dopamine reuptake inhibitors or precursors should provide further insight into these issues. A final explanation is that bromocriptine has different effects based on individual differences in the subjects’ dopaminergic systems, which together occlude any group effects (Cools and D’Esposito, 2011). While such an effect was not found when examined as a function of working memory or impulsivity, our sample size is insufficient to investigate individual differences based on genetic polymorphisms. This possibility warrants further investigation.

In our fMRI data, we have replicated and expanded the previous findings of Forstmann et al. (2008). We have confirmed that speeded decision making is associated with enhanced neural activation in the striatum and pre-SMA, as well as in the IPS, occipital pole, and posterior cingulate cortex. The observation that the occipital pole is also affected by speed cues is interesting, as it shows top-down effects on early perceptual processing regions, which take place before the target stimulus is presented. Effects of drift and threshold changes with SAT on activity in area V1 have recently been discovered (Ho et al., 2012). Understanding our fMRI data in relation to drug is not straightforward. At first glance, the cluster-corrected whole-brain maps (Figure 4A) suggest an effect in placebo and not in drug, but the difference between the two maps is not significant. This means that although one effect differs significantly from 0 and the other does not, the difference between the two cannot be interpreted statistically (Nieuwenhuis et al., 2011). Direct comparisons based on the activated regions would also not be a valid statistical analysis, since only including those voxels that show significant activation in one condition introduces a bias toward a significant difference between the conditions (Vul et al., 2009). Neither the whole-brain interaction between session and condition, nor the anatomical ROI analyses show a significant difference between drug and placebo, and Bayesian analyses show the evidence to be in favor of the null hypothesis. The absence of a difference is can be explained by the qualitative comparison of the non-thresholded images (Figure 4B). These show that there is subthreshold activation during drug in the regions that are significantly active during placebo. Taken together, our imaging findings suggest that there is no effect of bromocriptine on the effect of cue on the brain.

To summarize, we have replicated the finding that striatum and pre-SMA are active when preparing fast decisions. We have found that bromocriptine does not alter subjects’ decision threshold, and that it does not change the activation of the known speed related network in the brain.

## Conflict of Interest Statement

The authors declare that the research was conducted in the absence of any commercial or financial relationships that could be construed as a potential conflict of interest.
